# Research on creep characteristics of double fractured rock under freeze-thaw action

**DOI:** 10.1371/journal.pone.0320194

**Published:** 2025-05-14

**Authors:** Dengke Yang, Xiaoxiao Duan, Keyan Cheng, Lijun Xie, Yongjun Song

**Affiliations:** 1 School of Architecture and Civil Engineering, Xi ‘an University of Science and Technology, Xi’An, Shaanxi, China; 2 School of Intelligent Science and Engineering, Xi’an Peihua University, Xi’An, Shaanxi, China; 3 China Metallurgical Land Group Northwest Geotechnical Engineering Co., Ltd., Xi’an, Shaanxi, China; University of Sharjah, UNITED ARAB EMIRATES

## Abstract

Open rock masses are subject to prolonged loading and freeze-thaw cycles in cold regions. In this study, we take saturated fissured red sandstone as object to investigate the long-term mechanical response characteristics of fractured rock under freeze-thaw conditions. Experimental tests were conducted to analyze the creep characteristics of the rock after freeze-thaw cycles, considering different freeze-thaw frequencies and fracture orientations. The results reveal that (1) freeze-thaw cycles exert a significant influence on the rock’s creep behavior, with axial strain, instantaneous strain, and creep strain increasing progressively with the number of freeze-thaw cycles; (2) dual-fractured rock samples with varying fracture angles exhibit distinct differences in creep phenomena, where increased fracture angles result in pronounced increases in instantaneous and creep strains, and higher horizontal stress levels lead to greater strain generation; (3) all rock samples with different pre-existing fractures exhibit rock bridge breakthrough during creep failure, and the variation in fracture angle affects the failure mode; (4) and the long-term strength of the rock varies with changes in fracture angle and freeze-thaw cycle frequency, showing an increasing trend with greater fracture angles but a rapid decrease with increasing freeze-thaw cycles. These findings provide valuable insights for engineering design and risk assessment of open rock masses in cold regions. This study has guiding significance for the safety construction of rock mass engineering in cold regions.

## 1 Introduction

Creep has always been a focal point of research in rock engineering, with gravity-induced deformations persisting in the slope areas over time [1]. Moreover, rock bodies in cold regions frequently suffer from frost damage due to temperature variations, posing threats to structural stability and engineering construction [2]. Therefore, studying the creep characteristics of fissured rocks in freeze-thaw environments is of significant guidance for addressing stability issues in the construction of infrastructure projects in cold regions.

Most natural rock masses consist of intact rocks and different types of structural planes between them, including fissures, joints, and faults. Extensive research indicates that the presence of discontinuities such as fissures and joints affects the mechanical properties of the rock mass, reducing its strength [3]. In the cyclic diurnal and seasonal environments of cold regions, the water within the fissures undergoes freeze-thaw cycles, leading to phase transitions between water and ice. This process generates frost heave forces, further causing the development and interconnection of internal fissures in the rock mass, and ultimately leading to rock mass failure [4]. This paper investigates the red sandstone of a project in the Northern Shaanxi region as the research subject.

In the study of rock fractures, Debanjan et al. [5] conducted experimental research on the fracture and mechanical properties of water-saturated sedimentary rocks, comparing all the geomechanical and fracture properties of saturated rocks to explore their interrelationships. Farahmand, K., et al. [6] used a synthetic rock mass model combining Discrete Fracture Network (DFN) and Discrete Element Particle Model (DEM) to characterize the mechanical properties of medium-jointed rock masses under finite and infinite conditions. They discussed the importance of considering the geometric characteristics and interactions of fractures by simulating the mechanical behavior of jointed rock masses. Justo et al. [7], based on the Strain Energy Density (SED) criterion, thoroughly analyzed the fracture behavior of rocks under Mode I loading, pointing out that temperature is a key parameter in rock fracture characteristics, affecting the primary mechanical properties of rocks. Gell et al. [8] accurately simulated and studied the impact of specific fractures or defects on rock mechanical behavior using artificial samples, particularly three-dimensional printed samples, which helps to better understand the role of fractures in rock mechanical behavior. Cacace et al. [9]developed a theoretical framework and proposed advanced numerical simulation methods for the mechanical performance of fractured rocks, especially focusing on the role of elasto-plastic feedback in the rock deformation process, considering fractures as low-dimensional structures in load-bearing deformable porous rocks.

In the field of freeze-thaw research on fissured rocks, Park et al. [10] utilized X-ray computed tomography (CT) and scanning electron microscopy (SEM) to capture images of the internal microstructure of rocks, highlighting that internal volumetric expansion leads to increased porosity. Kolay [11] discussed the impact of freeze-thaw cycles on the engineering characteristics of sedimentary rocks, providing an effective method to assess and predict changes in rock performance during the freeze-thaw process through statistical analysis. Mutlutürk et al. [12] successfully described the integrity loss process of rocks under repeated freeze-thaw and temperature cycling by developing a mathematical model, which was validated experimentally for its applicability and accuracy. Mousavi [13] investigated the textural and microstructural changes of samples after weathering using scanning electron microscopy (SEM), delving into how freeze-thaw cycles affect the engineering characteristics of limestone and offering a practical empirical model for predicting and evaluating the performance of such rocks under similar conditions. Fakhri et al. [14] developed a technique based on Gaussian process regression (GPR) to predict the failure load of rock samples under various freeze-thaw (F-T) cycle conditions, identifying confining pressure and porosity as the main factors influencing the failure load, thereby laying the groundwork for accurately estimating the failure load of rock samples under F-T cycles. Yahaghi et al. [15] studied the physico-mechanical properties and failure behavior of sandstone under different freeze-thaw cycles through experimental and three-dimensional (3D) numerical modeling methods, revealing the degradation and failure mechanisms of sandstone under varying freeze-thaw cycles. Aral et al. [16] analyzed the water absorption rate, P-wave velocity, uniaxial compressive strength, point load strength index, and Schmidt hammer hardness value through experimental analysis to quantitatively evaluate the disintegration rate of rocks under the influence of freeze-thaw cycles.

Beyond the effects of freeze-thaw cycles, another critical factor in engineering construction is the potential for creep under long-term loading of rock samples. Gutiérrez-Ch et al. [17] employed Rate Process Theory (RPT) in conjunction with the Discrete Element Method (DEM) to simulate creep deformation in rocks within deep tunnels, offering an effective approach to understanding and modeling time-dependent deformation in such contexts. W Frenelus [18] reviewed factors influencing rock creep and conducted a thorough study on the mechanisms of creep fracture in rocks. Cirone et al. [19] provided a model development framework based on the principles of continuum thermodynamics to describe the creep behavior of rock salt, introducing a dual-mechanism model for transient and steady-state creep, which serves as a crucial theoretical and modeling tool for understanding and predicting the creep behavior of rock salt in long-term applications. Taheri et al. [20] conducted triaxial creep experiments on rock salt samples under various temperatures and differential stress conditions, revealing specific creep characteristics of rock salt that are essential for preventing and managing casing collapse issues in oil fields. Delchiaro et al. [21] adopted a multi-perspective approach to explore the dynamic feedback between surface processes, tectonic actions, and time-dependent rock deformation, aiding in predicting landslide hazards in areas prone to large-scale rock slope instabilities. Discenza et al. [1] considered the viscoplastic behavior of jointed rock masses as a primary topic in creep studies and experimentally demonstrated the significant impact of extensive jointing on rock mass creep at the slope scale. Tarifard et al. [22] examined the effects of rock creep behavior and groundwater on the long-term stability of tunnels, noting that the creep behavior of weak rocks and the impact of groundwater, especially in tunnel spring lines and fault zones, significantly influence tunnel stability. Sabitova et al. [23] studied the response of rocks during creep and stress relaxation tests, identifying different stress-strain rate behaviors, which are crucial for understanding the natural deformation processes of rocks over geological time scales and their behavior in subsurface engineering operations. These findings are of significant reference value for the long-term stability of geotechnical engineering.

However, research on cold region rock engineering has primarily focused on mechanical tests of intact rocks and freeze-thaw damage models [24], with less attention to the creep characteristics of fissured rocks during freeze-thaw cycles and inadequate consideration of the interactions between multiple fractures. Therefore, it is essential to thoroughly investigate the impact of freeze-thaw actions on the long-term stability of fissured rocks and explore the effects of fractures and freeze-thaw actions on the creep characteristics of rocks, providing technical support for the construction of fissured rock body engineering. This study also fills the gap in research on the creep characteristics and long-term strength effects of fractured rock masses in freeze-thaw environments. In the next phase, the uniqueness of fractured rock masses should be considered in the development and construction of projects in cold regions, and proactive measures such as disaster assessments or grouting operations can be implemented to mitigate potential risks and losses.

## 2 Materials and methods

### 2.1 Sample preparation

Engineering practice and relevant literature indicate that discontinuous fractured rock masses are more common in rock engineering. This is particularly evident in slope engineering, where under the combined action of gravitational forces on the slope mass and external loads, rock bridges connecting fractures can undergo coalescence, ultimately leading to instability and failure of the rock mass [25]. The generation of fracture orientations in naturally occurring fractured rock masses lacks a clear pattern, making it challenging to simulate with pre-fabricated rock samples. However, fractures can be categorized based on the inclination angle into gentle (0°45°) and steep (45°90°), and combinations of these angles can be used to explore the mutual influence between fractures.

**Fig 1:**
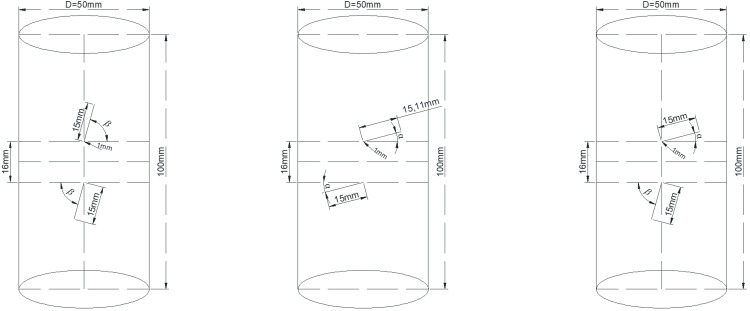
Schematic diagram of rock samples.

Therefore, for this experiment, we selected fracture inclinations of 15° (gentle) and 75° (steep), combining them in various configurations to simulate the interactions between different types of fractures [26]. Two parameters, α and β, were used to control the geometric positions of the two pre-existing fractures. The three angle combinations were named gentle-gentle, gentle-steep, and steep-steep based on the magnitudes of α and β. Simultaneously, an intact rock sample was set as the reference group. The schematic representation of the rock samples is illustrated in Fig 1. (α=15°, β=75°).

The raw material for sample preparation consisted of homogenous and intact red sandstone. Following the methods recommended by the International ISRM testing procedures, the samples were processed into standard cylindrical shapes with dimensions of 50mm × 100mm (diameter × length). Both ends of the samples were ground flat, ensuring a parallelism within 0.1mm. Subsequently, pre-set fractures, including their angles, lengths, and positions, were created by using a high-speed water jet to cut two penetrating fractures in the middle of the samples. The fractures had a length of 15.0mm, a width of 1.0mm, and the rock bridge length was set at 16.0mm.

Referring to the standards of engineering rock testing methods, visually distinct rock samples were excluded before the experiment. The remaining samples were placed in an oven at a constant temperature of 105°C for 24 hours. After cooling, the dry density of the samples was measured. The samples were then vacuum-saturated at 0.1MPa for 4 hours, followed by a 4-hour static period under atmospheric pressure. Afterward, the saturated mass of the samples was measured, providing the mean values of basic physical parameters for each group of rock samples, as summarized in Table 1.

**Table 1:** Mean values of physical parameters of rock samples.

uniaxial compressive strength /(MPa)	dry density /(g·cm^-3^)	saturation density /(g·cm^-3^)	percentage of saturated water content /%	porosity /%
19.57	2.23	2.36	6	13.35

### 2.2 Test scheme and process

To comprehensively study the creep characteristics of fractured rock under freeze-thaw conditions, a series of experiments were designed, including freeze-thaw cyclic tests, uniaxial compression tests, and creep tests. The freeze-thaw cyclic tests aimed to simulate the alternating freezing and thawing of rocks to replicate cold climate conditions. The loading conditions were set, and the mechanical behavior and deformation characteristics of the rocks under the corresponding conditions were observed. Subsequently, the rock creep characteristics were investigated. Following the standards of engineering rock testing methods, the schemes and procedures for freeze-thaw cyclic tests, uniaxial compression tests, and creep tests were determined as follows.

1) Freeze-thaw cyclic tests

Place all prepared rock samples, except for the ones subjected to zero freeze-thaw cycles, into the freeze-thaw cyclic testing chamber. Conduct the freeze-thaw cyclic tests to simulate the conditions of alternating freezing and thawing, mirroring actual conditions in cold regions. Following the standards of engineering rock testing methods and considering the practical conditions in cold regions, set each freeze-thaw cycle period to 24 hours, with 12 hours of freezing and 12 hours of thawing. The temperature for freeze-thaw cycles is maintained at ±20°C. Depending on the specific experimental requirements and the observed impact of freeze-thaw cycles on the physical-mechanical properties of rocks during pre-tests, set the number of freeze-thaw cycles at 0, 30, 60, and 90 cycles.

2) Uniaxial Compression Tests after Freeze-Thaw Cycles

The experimental loading equipment employed is the TW-1000 rock mechanics testing machine, which has a maximum loading capacity of 1000 kN. It is computer-controlled, allowing real-time recording of load, stress, strain, and displacement. In the uniaxial compression tests conducted on rock samples subjected to different freeze-thaw cycle numbers and fracture inclinations, the loading method follows deformation control with a rate of 0.05 mm/min. Mechanical data is collected at a frequency of 2 samples/second until the rock samples undergo failure.

3) Creep test

**Fig 2:**
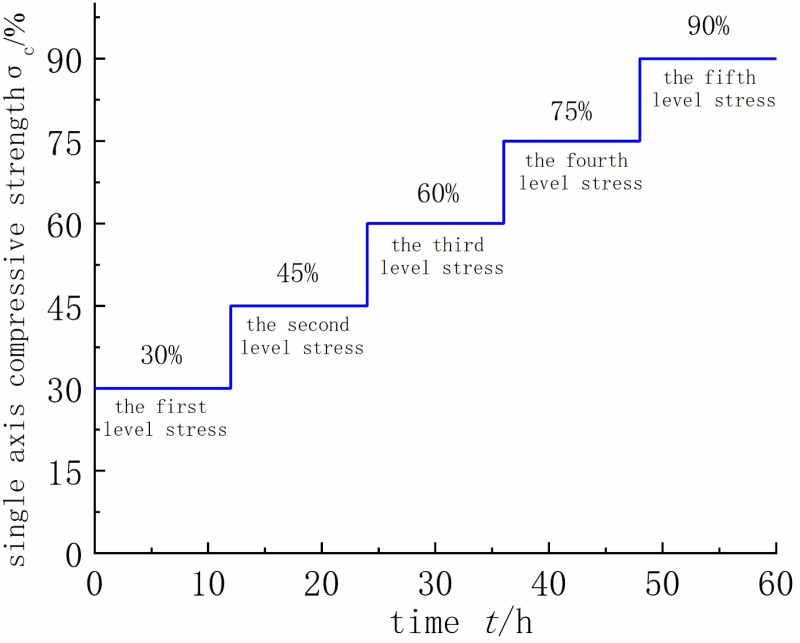
Graded loading creep stress loading level.

**Fig 3:**
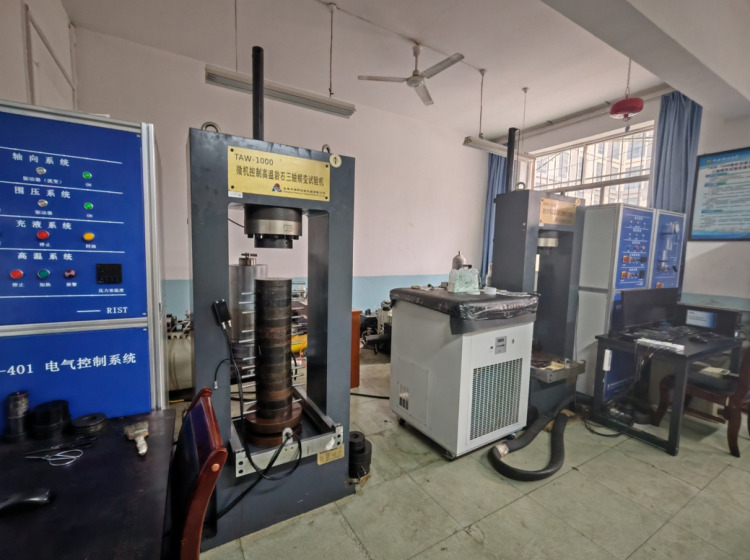
Rock mechanics testing machine.

This experiment employed a staged loading creep test method, subjecting the four types of rock samples to staged loading creep tests. The uniaxial compressive strength of the intact rock sample was measured at 19.57 MPa. In this experiment, the loading stress for the first stage was set at 30% of the uniaxial compressive strength. Subsequently, each stage increased by 15% until failure. The stress levels for stages one to five were 30%, 45%, 60%, 75%, and 90%, respectively. If, under the fifth stage creep stress loading, the rock sample did not undergo failure, the sixth stage loading stress was increased by only 10% compared to the previous stage, set at 100%. The specific stress loading levels for each stage are illustrated in Fig 2.During the creep loading, the loading speed was set at 0.01 MPa/s, with each load stage lasting for 12 hours before proceeding to the next stress level, repeating the process sequentially. The test operation platform is shown in Fig 3.

In the testing process, the study subjected rock samples to loading through uniaxial and creep tests to investigate the deformation and failure mechanisms of rocks under different stress conditions. At the initial stage of loading, the rock exhibits elastic deformation, with no significant physical changes in the sample. As the stress approaches the rock’s compressive strength, microcracks begin to appear on the sample surface and gradually propagate inward. With continued loading, the rock enters the plastic deformation stage, where cracks further propagate, leading to the bridging of cracks and ultimately resulting in failure. However, the influence of various factors on rock failure still necessitates a more comprehensive analysis.

## 3 Results

### 3.1 Effect of freeze-thaw cycles on creep properties of rock

**Fig 4:**
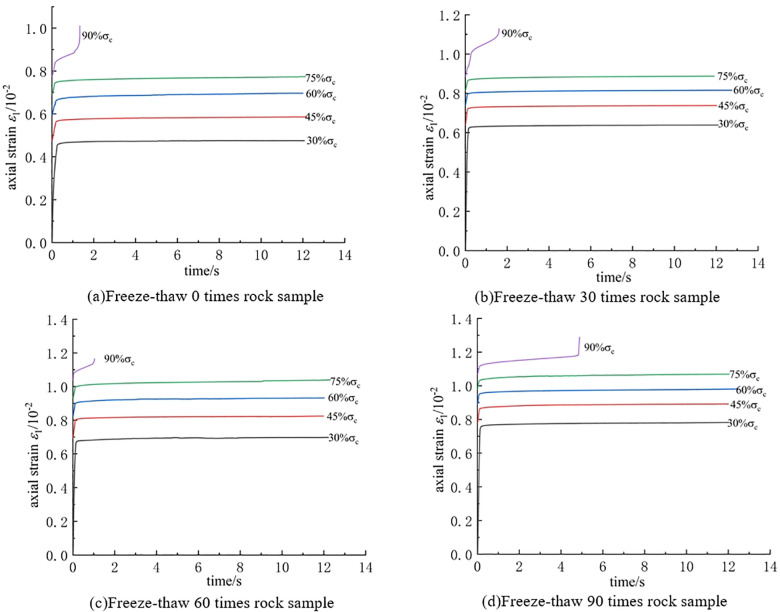
Clusters of creep curves under different freeze-thaw cycles. **(a)** Rock sample without freeze-thaw cycles. **(b)** Rock sample after 30 freeze-thaw cycles.**(c)** Rock sample after 60 freeze-thaw cycles.**(d)** Rock sample after 90 freeze-thaw cycles.

To investigate the impact of freeze-thaw cycles on the creep characteristics of rocks, the creep curves of intact rock samples after different numbers of freeze-thaw cycles (0 cycles, 30 cycles, 60 cycles, 90 cycles) were analyzed. The Chen’s loading method was applied to transform the curves into staged loading creep curves, as illustrated in Fig 4. Fig 4 presents the staged loading creep curves of the intact rock sample after 0, 30, 60, and 90 freeze-thaw cycles. From the curves, it is evident that freeze-thaw cycles have a significant effect on the deformation of the rock samples. As the stress loading level increases, the axial strain of the rock sample gradually increases until failure. Additionally, with an increasing number of freeze-thaw cycles, the axial strain at the point of ultimate failure becomes larger, and the total creep deformation markedly increases.

1) **The effect of freeze-thaw cycles on the instantaneous strain of rock samples**

**Fig 5:**
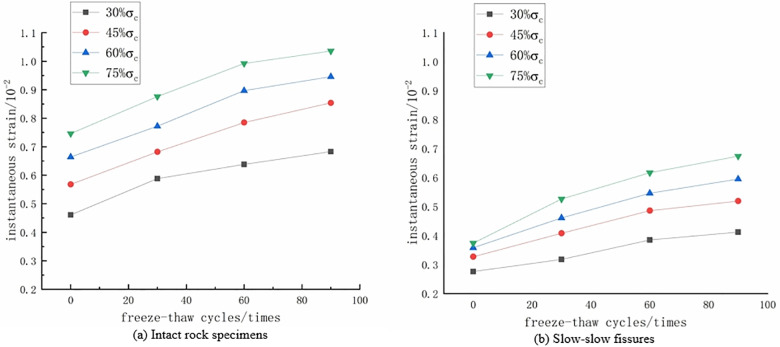
The variation of instantaneous strain with the number of freeze-thaw cycles. **(a)** Intact rock specimens. **(b)** Slow-slow fissures.

As shown in Fig 5, the instantaneous strain of the intact rock sample and the gentle-gentle fracture-angled rock sample varies at the first four stress levels under different freeze-thaw cycle numbers. Observing Fig 5 reveals that, at the same stress level, the instantaneous strain of the rock sample significantly increases with an increase in the number of freeze-thaw cycles. However, the rate of increase gradually diminishes and tends to stabilize. Simultaneously, with an increase in the number of freeze-thaw cycles, the influence of stress levels on the instantaneous strain generated in the rock sample becomes more pronounced.

2) **Effect of freeze-thaw cycles on creep strain of rock samples**

**Fig 6:**
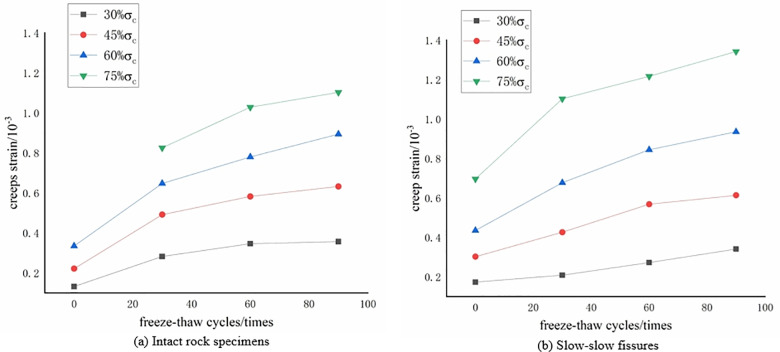
The variation of creep strain with the number of freeze-thaw cycles. **(a)** Intact rock specimens. **(b)** Slow-slow fissures.

As illustrated in Fig 6, the variations in the creep strain of the intact rock sample and the gentle-gentle fracture-angled rock sample at different stress levels with varying freeze-thaw cycle numbers are presented. It is noteworthy that, due to a sudden increase in axial strain observed in the zero freeze-thaw cycle gentle-gentle rock sample during the fourth stage loading, it is not reflected in the graph. Fig 6 shows that regardless of the presence of fractures, both rock samples exhibit noticeable creep strain from the first stage loading, and this creep strain increases with the number of freeze-thaw cycles. This indicates a pronounced enhancement of creep strain in the rock samples due to freeze-thaw cycles.

3) **The effect of freeze-thaw cycles on the steady creep rate of rock samples**

**Fig 7:**
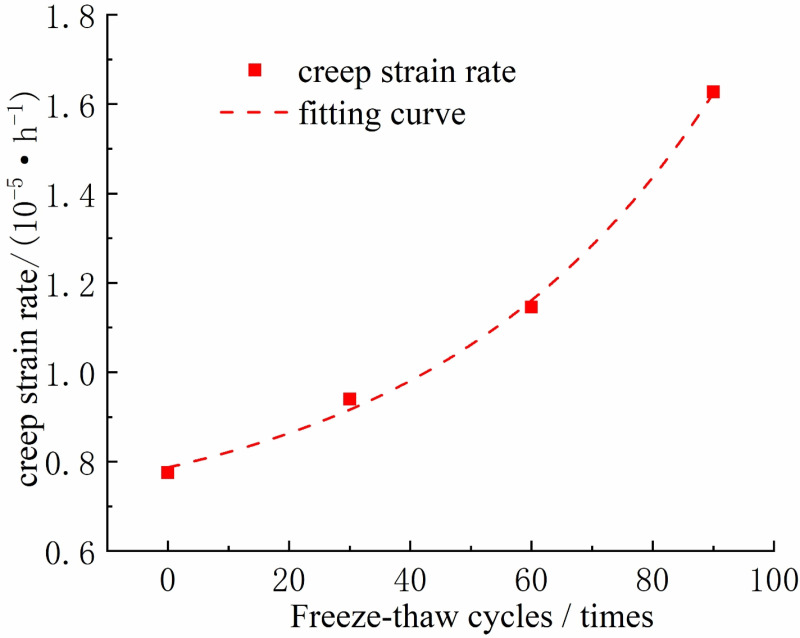
Variation of creep strain rate of rock samples with the number of freeze-thaw cycles.

As depicted in Fig 7, the steady-state creep rate of the intact rock sample at the third-stage loading stress level under different freeze-thaw cycle numbers is presented. Through the analysis of Fig 7, it can be observed that when the rock sample has not undergone any freeze-thaw cycles, its steady-state creep rate is 7.757×10^-4^. However, after 30 freeze-thaw cycles, the steady-state creep rate increases by 21.12%, reaching 9.396×10^-4^. With an increase in the number of freeze-thaw cycles, the steady-state creep rate of the rock sample continues to grow, and the growth rate accelerates. Therefore, as the number of freeze-thaw cycles increases, the steady-state creep rate of the rock sample also increases.

Fitting the experimental results, as shown in Fig 7, provides an excellent description of the relationship between the number of freeze-thaw cycles and the steady-state creep rate of the rock sample. The relevant fitting equation is shown in formula (1):


$$\varepsilon_{c}\;=\;6.42692\;\times \;10^{\text{-}4}\;+\;1.44383\;\times \;10^{\text{-}4}e^{\frac{N}{46.95788}},\;R^{2}\;=\;0.99776$$ (1)

Where, $\varepsilon_{c}$ is the steady state creep rate, and N is the number of freeze-thaw cycles.

### 3.2 Creep characteristics of rock under different fracture dip angles

**Fig 8:**
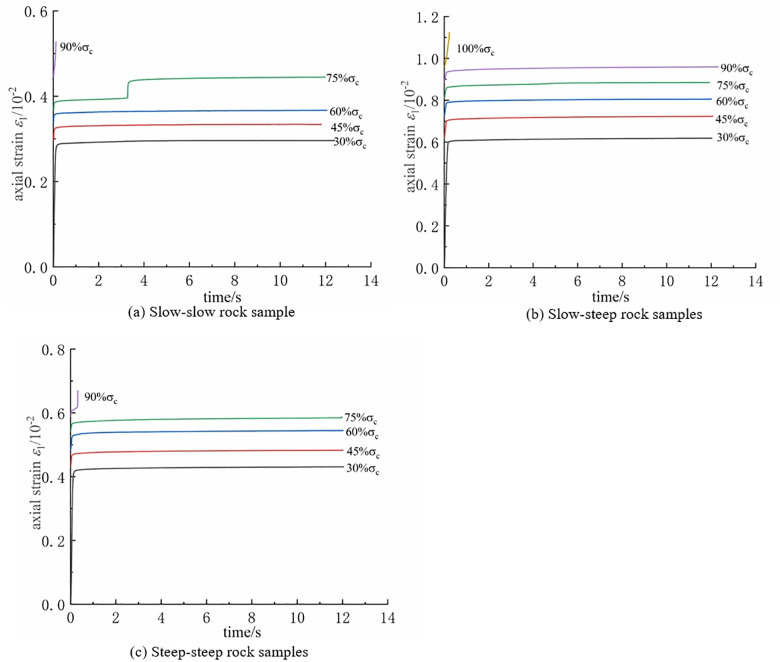
Creep curve clusters of rock samples with different fracture dip angles. **(a)** Slow-slow rock sample. **(b)** Slow-steep rock samples. **(c)** Steep-steep rock samples.

Applying the Chen’s loading processing method, the creep curves under different stress levels were obtained for rock samples with various fracture angles without undergoing freeze-thaw cycles. The data on the right side of the graph represents the stress levels at each stage. From Fig 8 Creep curve clusters of rock samples with different fracture dip angles, it can be observed that, during the fourth stage loading at the gentle-gentle fracture-angled rock sample, a sudden increase in axial strain occurred at 4.1 hours. The main reason for this sudden strain increase is the occurrence of breakthrough in the rock bridge connecting the two prefabricated fractures under creep stress loading, leading to a sudden increase in axial strain. Additionally, it can be observed that all types of rock samples exhibit distinct stages of constant-rate creep and decelerated creep. As the stress loading level increases, the total strain at each stage significantly increases. This indicates that, compared to the other two types of rock samples, the steep-steep fracture-angled rock sample experiences less weakening of its mechanical performance during creep.

1) **The influence of fracture dip angle on the instantaneous strain of rock samples**

**Fig 9:**
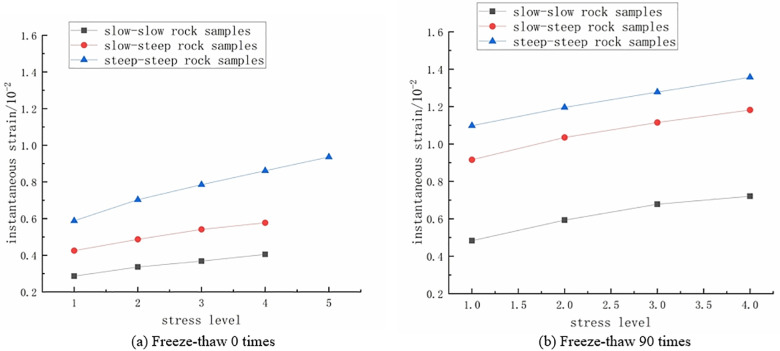
The variation of instantaneous strain of rock samples with different dip angles of double fissures with stress level. **(a)** Freeze-thaw 0 times. **(b)** Freeze-thaw 90 times.

As shown in Fig 9, the instantaneous strain of the three types of double-fractured rock samples varies under different stress levels at freeze-thaw cycle numbers of 0 and 90. When the freeze-thaw cycle number is 0, the instantaneous strains of the gentle-gentle, gentle-steep, and steep-steep rock samples at the first stress level (30%) are 0.283×10^-2^, 0.415×10^-2^, and 0.595×10^-2^, respectively. Compared to the gentle-gentle rock sample, the instantaneous strains of the gentle-steep and steep-steep rock samples increased by 46.64% and 110.24%, respectively. Therefore, at the same stress level, the instantaneous strain of the fractured rock samples shows a noticeable increasing trend with the increase in fracture angle, and the instantaneous strain linearly increases with the stress level.

2) **The influence of crack dip angle on creep strain of rock specimen**

**Fig 10:**
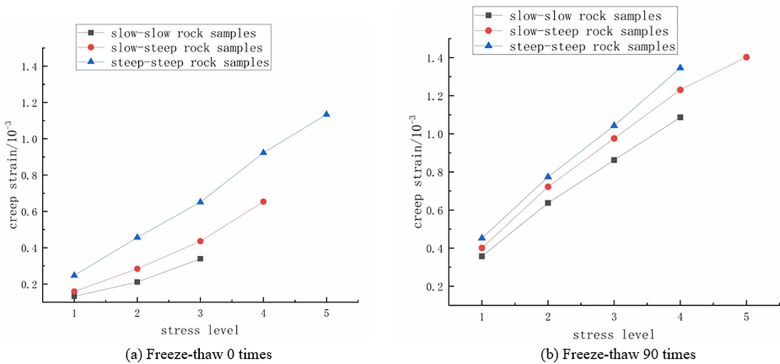
The variation of creep strain of rock samples with different dip angles of double fissures with stress level. **(a)** Freeze-thaw 0 times. **(b)** Freeze-thaw 90 times.

As depicted in Fig 10, the variations in creep strain for the three types of fractured rock samples at freeze-thaw cycle numbers of 0 and 90 under different stress levels are presented. From the graph, it can be observed that, at the same stress level, the steep-steep rock sample exhibits the highest creep strain, while the gentle-gentle rock sample shows the lowest creep strain. This indicates that creep strain increases with the increase in fracture angle, and the variation in fracture angle significantly affects creep strain. Additionally, within the same rock sample, creep strain also significantly increases with the rise in stress level. Therefore, at the same stress level, creep strain increases with the increase in fracture angle, and the increase is more pronounced with higher stress levels.

3) **The influence of fracture dip angle on steady-state creep rate of rock samples**

Table 2 provides the steady-state creep rates for the three types of unfrozen fractured rock samples at the third stress level.

**Table 2:** Steady-state creep rate of rock samples under different crack dip angles.

Rock type	Slow-slow rock sample	Slow-step rock sample	Step-step rock sample
steady creep rates/(10^–5^·h^-1^)	0.485	0.976	0.991

Based on the data in Table 2, it can be observed that the steady-state creep rate for the gentle-gentle fractured rock sample is 4.85×10^-4^, for the gentle-steep fractured rock sample it is 9.76×10^-4^, and for the steep-steep fractured rock sample it is 9.91×10^-4^. It is evident that among the three types of fractured rock samples, the steep-steep fractured rock sample exhibits the highest steady-state creep rate, while the gentle-gentle fractured rock sample has the lowest steady-state creep rate. This indicates that as the fracture angle increases, the steady-state creep rate also increases. This is attributed to the fact that an increase in fracture angle enhances the uniaxial compressive strength of the rock sample. Consequently, the higher uniaxial compressive strength at the same stress level leads to an increase in applied loading stress, resulting in an elevated creep rate.

### 3.3 Creep failure characteristics and test results of rock

1) **Creep failure mode of rock**

**Fig 11:**
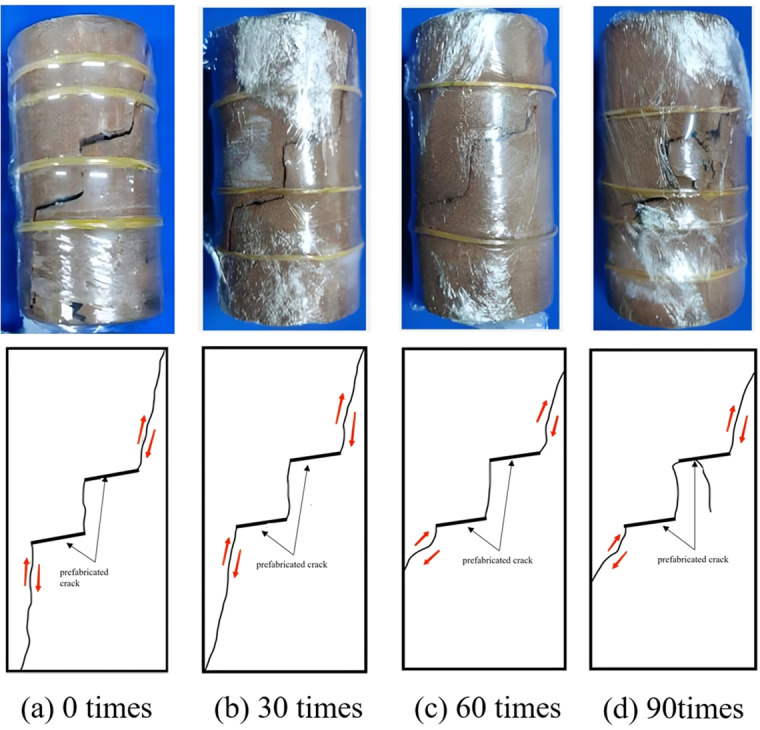
Creep failure mode and sketch map of slow-slow fractured rock. **(a)** 0 times. **(b)** 30 times. **(c)** 60 times. **(d)** 90 times.

**Fig 12:**
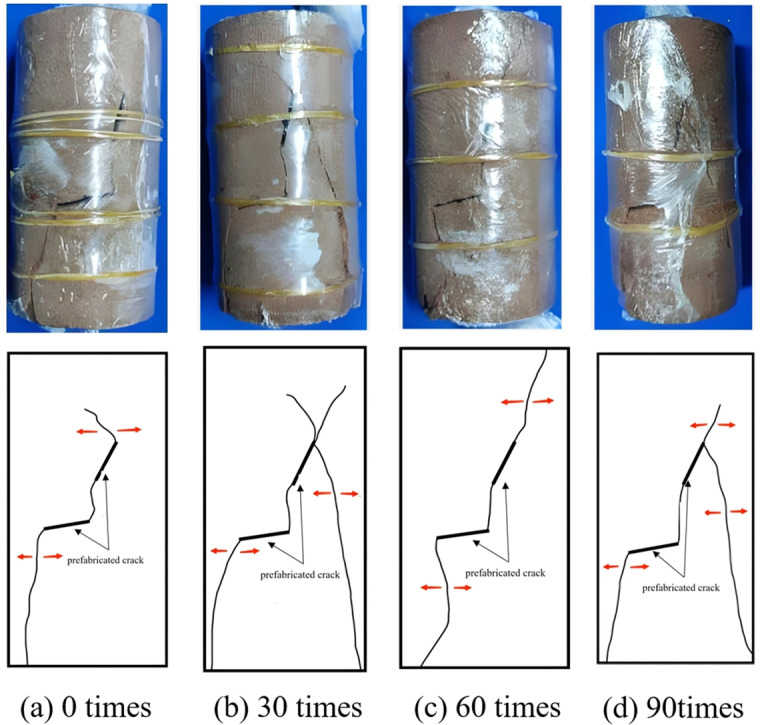
Creep failure mode and sketch map of slow-step fractured rock. **(a)** 0 times. **(b)** 30 times. **(c)** 60 times. **(d)** 90 times.

**Fig 13:**
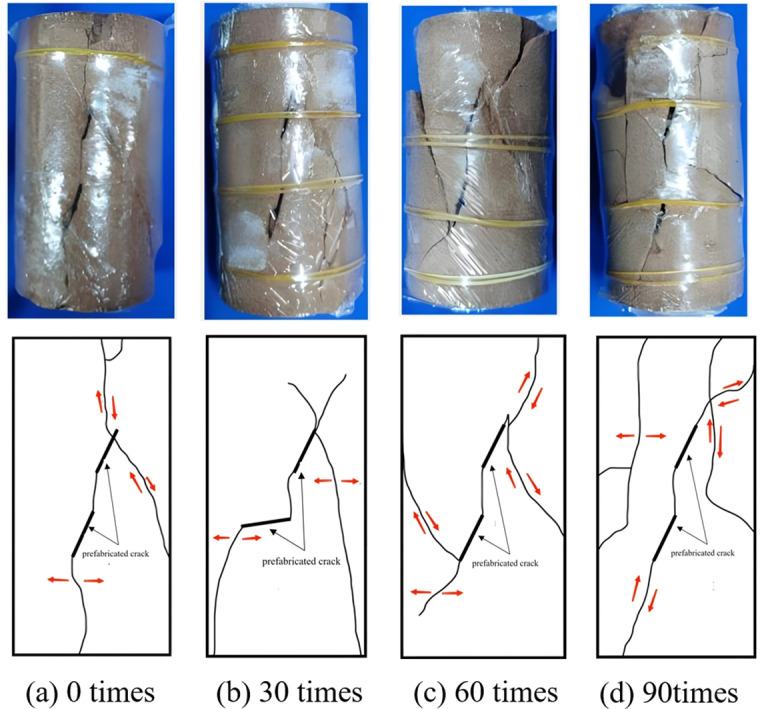
Creep failure mode and sketch map of step-step fractured rock. **(a)** 0 times. **(b)** 30 times. **(c)** 60 times. **(d)** 90 times.

**Fig 14:**
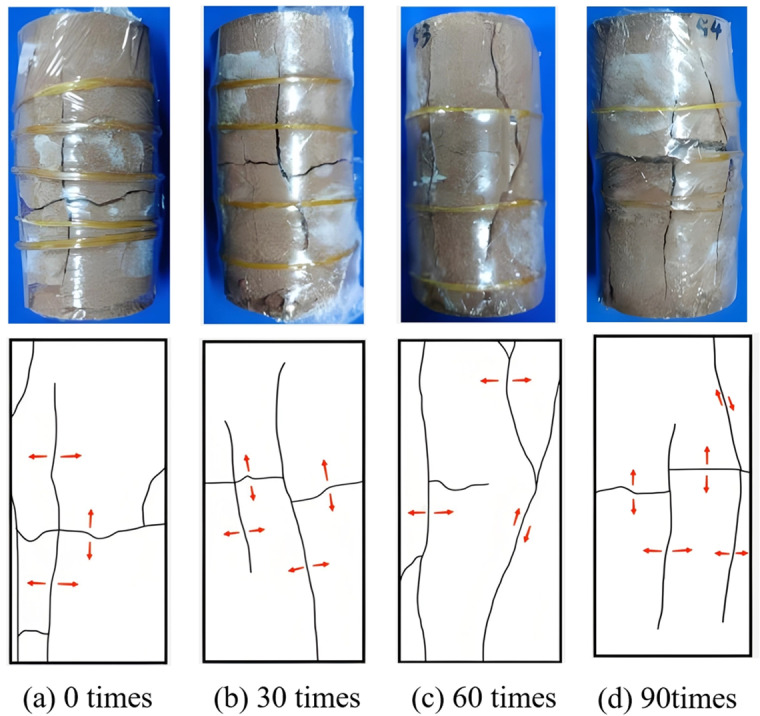
Creep failure morphology and sketch map of intact rock. **(a)** 0 times. **(b)** 30 times. **(c)** 60 times. **(d)** 90 times.

As depicted in Figs 11–14, the creep failure morphology and sketches of various fractured rock samples under different freeze-thaw cycles are presented. Fig 11 reveals that under graded creep loading conditions, the gentle-gentle fractured rock sample’s cracks are mainly composed of two shear cracks. The rock bridge between the two pre-existing fractures eventually undergoes penetration, resulting in shear-type failure. As shown in Fig 12, under graded creep loading conditions, the cracks in the gentle-steep fractured rock sample are caused by the extension of wing cracks. These cracks are tension cracks, and the rock bridge between the pre-existing fractures undergoes penetration, resulting in a tensile failure mode. Through analysis of Fig 13 it can be observed that under graded creep loading conditions, at different freeze-thaw cycles, the cracks in the steep-steep rock sample include both tension cracks and shear cracks. Meanwhile, the rock bridge undergoes penetration, resulting in a tensile-shear mixed failure mode. Fig 14 illustrates the creep failure morphology of the intact rock sample under different freeze-thaw cycles, and the failure mode is a tensile-shear mixed failure mode.

In summary, for pre-existing fractured rocks, regardless of the fracture angle of the rock sample, the failure process will exhibit rock bridge penetration. However, the failure modes of the three fractured rock samples are different. The gentle-gentle fractured rock sample primarily exhibits shear cracks, with a failure mode of shear failure. The gentle-steep fractured rock sample mainly exhibits tension cracks, with a failure mode of tensile failure. The steep-steep fractured rock sample shows both tension and shear cracks, with a failure mode of tensile-shear mixed failure. The failure mode of the intact rock sample transitions from tensile failure to tensile-shear mixed failure as the number of freeze-thaw cycles increases. Additionally, the freeze-thaw cycling exacerbates surface cracking and increases the number of failure cracks.

2) **Graded loading creep test results**

**Fig 15:**
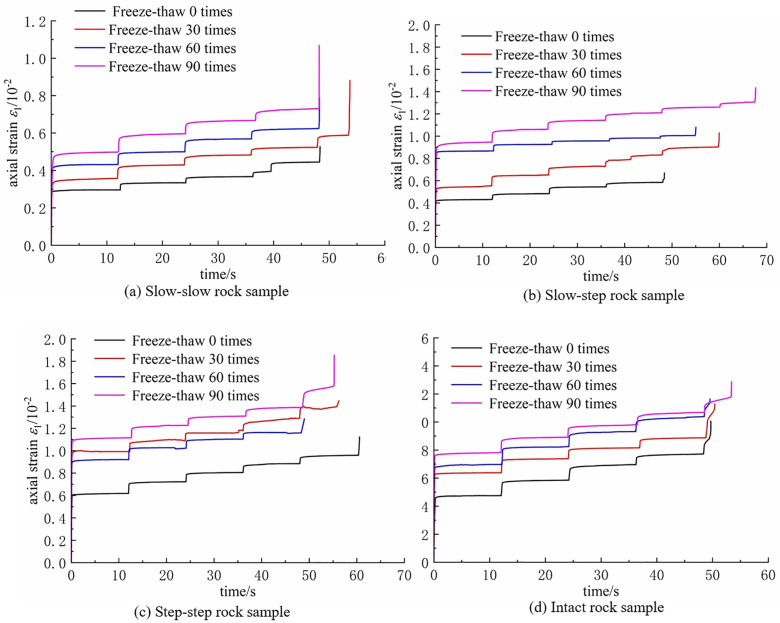
Relationship between axial strain and time curve. **(a)** Slow-slow rock sample. **(b)** Slow-step rock sample. **(c)** Step-step rock sample. **(d)** Intact rock sample.

As displayed in Fig 15, the creep curves of different types of double-fractured rock samples under graded loading at different freeze-thaw cycle numbers are presented.

From the figure, it can be observed that freeze-thaw cycles have a significant impact on rock creep deformation. For the same type of rock sample, the axial strain of the rock significantly increases with the number of freeze-thaw cycles. This is because, under the influence of freeze-thaw cycles, water in the fractures undergoes repeated freezing and expansion, leading to the extension of microcracks and damage in the rock sample, thereby causing an increase in creep deformation. At the same time, the figure also shows that some rock samples, especially those with a gentle-gentle fracture angle, undergo failure during the creep stress loading process but do not exhibit an accelerated creep stage.

### 3.4 Long-term creep strength of fractured rock under freeze-thaw action

The long-term strength of rocks refers to the critical stress threshold at which rocks transition from steady-state creep (characterized by a stable creep rate with a curve approaching a straight line) to non-steady-state creep (exhibiting an increasing or decreasing creep rate) during the creep process. In essence, when the applied creep stress on the rock is below the critical stress value, the rock undergoes steady-state creep, characterized by prolonged deformation without failure. Conversely, when the applied creep stress exceeds the critical stress, non-steady-state creep occurs, eventually leading to creep failure of the rock. Hence, the long-term strength of rocks plays a pivotal role in determining the safety and stability of rock engineering projects, making it a focal point in the study of rock creep behavior.

The methods for determining the long-term strength of rocks include the transitional creep method and the isochronous curve method, each of which has certain inaccuracies in calculating the long-term strength of rocks. To reduce these errors in calculating the long-term strength of rocks, this study employs the tangent method to the steady-state creep rate curve. By analyzing the relationship between steady-state creep rate and axial stress under various stress levels, it is observed that before the critical turning point, the steady-state creep rate of the rock increases slightly with the level of deviatoric stress, but the increment is very small. After the critical turning point, the curve rises sharply, indicating that the rock has entered the unstable creep stage. Two tangents are drawn to the curve before the critical point, and the axial stress corresponding to their intersection is determined as the long-term strength of the rock.

**Fig 16:**
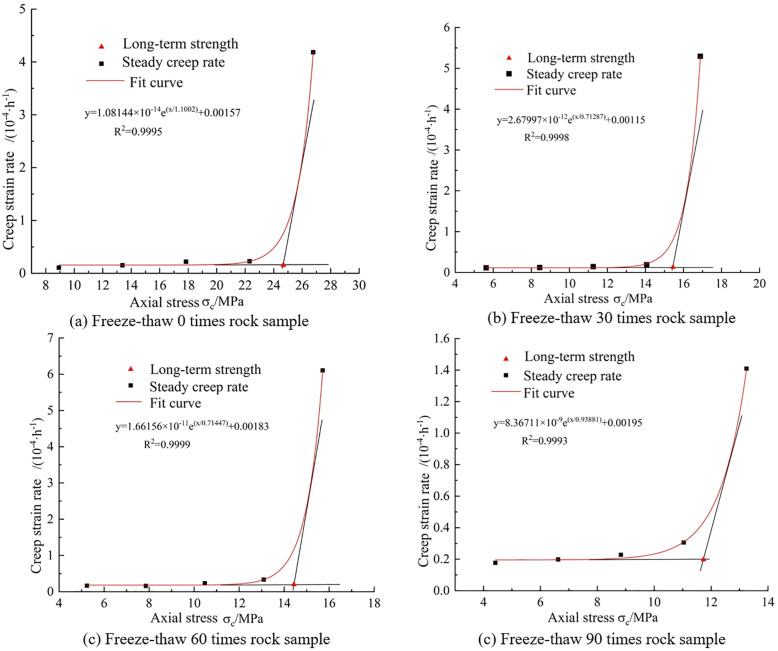
Creep long-term strength of intact rock samples under different freeze-thaw cycles. **(a)** Freeze-thaw 0 times rock sample. **(b)** Freeze-thaw 30 times rock sample. **(c)** Freeze-thaw 60 times rock sample. **(d)** Freeze-thaw 90 times rock sample.

As illustrated in Fig 16, the long-term strength of intact rock samples subjected to varying numbers of freeze-thaw cycles is presented. The graph reveals that, after experiencing 0, 30, 60, and 90 freeze-thaw cycles, the long-term strength of the intact rock samples is 24.676 MPa, 15.449 MPa, 14.432 MPa, and 11.734 MPa, respectively. In comparison to the rock samples that did not undergo freeze-thaw cycles, those subjected to 30, 60, and 90 cycles exhibit a decrease in long-term strength by 9.227 MPa, 10.244 MPa, and 12.942 MPa, respectively. It is evident that the long-term strength of rocks significantly decreases with an increase in the number of freeze-thaw cycles.

**Fig 17:**
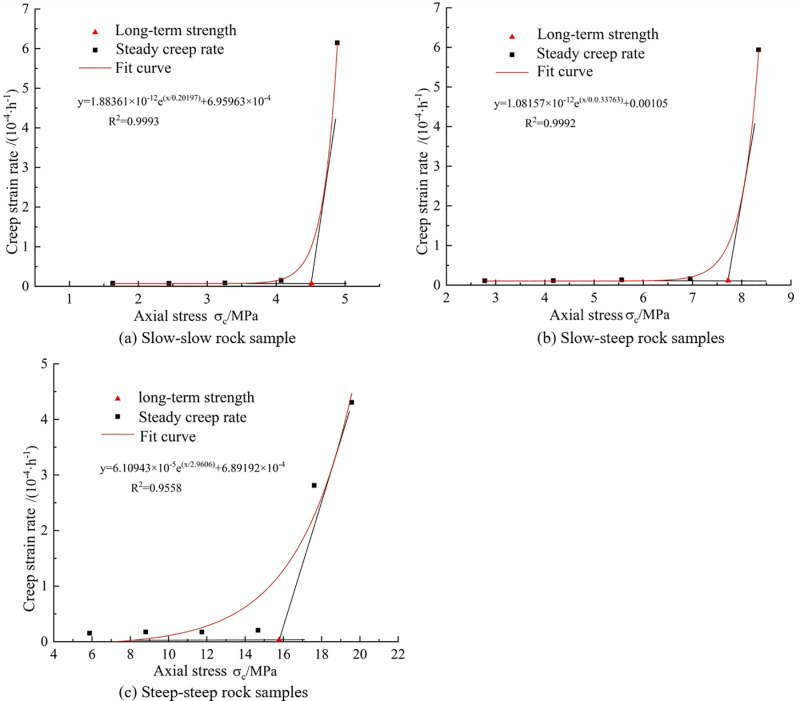
Long-term creep strength of three kinds of double-fractured rock samples. **(a)** Slow-slow rock sample. **(b)** Slow-steep rock samples. **(c)** Steep-steep rock samples.

As presented in Fig 17, the long-term strength of three types of rock samples without undergoing freeze-thaw cycles is shown. As depicted in the graph, for the rock samples that did not experience freeze-thaw cycles, the long-term strength of the gentle-gentle rock sample is 4.512 MPa, the long-term strength of the gentle-steep rock sample is 7.721 MPa, and the long-term strength of the steep-steep rock sample is 15.778 MPa. Comparing these values with the intact rock sample, it is observed that the long-term strength of these rock samples has decreased by 81.72%, 68.71%, and 36.06%, respectively. This indicates that a reduction in fracture angle leads to a rapid decline in the long-term strength of rock samples, emphasizing the significant impact of fractures on the long-term strength of rocks. Therefore, in practical rock engineering, special attention should be given to the influence of fractures on rock mass behavior.

## 4 Disscusion

### 4.1 The impact of freeze-thaw cycles on the failure behavior of rock masses

Freeze-thaw cycling is one of the most prominent external environmental factors considered in this study. The repeated action of freeze-thaw cycles causes moisture within the rock mass to expand and contract at fracture sites, thereby accelerating fracture propagation and rock failure [27]. The study shows that freeze-thaw cycles significantly affect the creep behavior and long-term strength of rock masses [28], as detailed below:

Freeze-thaw cycles notably enhance both the instantaneous strain and creep strain of rock masses under the same stress level. As the number of freeze-thaw cycles increases, the creep rate accelerates, resulting in greater total creep deformation(Fig 4-6). This indicates that freeze-thaw actions enhance the deformation capacity of rock masses, making them more prone to plastic deformation and failure under long-term loading. The repeated freeze-thaw process promotes the propagation of microcracks within the rock mass, which results in higher stress concentration zones, thereby accelerating the creep process [29].

Freeze-thaw cycles lead to a substantial decline in the long-term strength of rock masses. Research findings indicate that after undergoing 90 freeze-thaw cycles, the long-term strength of the rock mass decreased by nearly 12.94 MPa, showing a strong inverse relationship with the number of freeze-thaw cycles. This reduction is primarily due to changes in the microstructure of the rock mass caused by freeze-thaw actions, which further exacerbate fracture propagation and diminish the rock’s resistance to external forces.

The cumulative effects of freeze-thaw cycles indicate that rock masses undergo continuous degradation in freeze-thaw environments, particularly for water-bearing fractured rocks, with a more pronounced failure process. Therefore, the impact of freeze-thaw cycles on the stability and safety of rock masses should be given special attention in rock engineering projects in cold regions.

### 4.2 The impact of fracture angles on the failure behavior of rock masses

Fracture angle is another critical factor influencing the mechanical behavior of fractured rock masses. By comparing the creep characteristics and failure modes of rock masses with varying fracture angles, this study reveals the significant impact of fracture angles on the failure behavior of rock masses. It was found that changes in fracture angles affect the failure modes, strain characteristics, and long-term strength of rock masses [30].

An increase in fracture angle notably exacerbates the creep deformation of rock masses. Under the same stress level, rock masses with steeper fracture angles exhibit higher instantaneous strain and creep strain. This is because increasing the fracture angle alters the internal force transmission and stress distribution within the rock mass, thereby weakening the rock structure and enhancing its deformation capacity under loading. Additionally, larger fracture angles reduce the stability of the rock mass, leading it to enter an unstable creep stage at lower stress levels(Fig 7-9).

Fracture angles profoundly affect the failure modes of rock masses [31]. For rock masses with smaller fracture angles (e.g., gentle-gentle fracture angles), the primary failure mode is shear failure. In contrast, rock masses with larger fracture angles (e.g., gentle-steep fracture angles) tend to exhibit tensile failure. As the fracture angle further increases, the failure mode progressively evolves into a mixed tensile-shear failure. This change indicates that larger fracture angles alter the propagation and connection of cracks within the rock mass, thereby affecting its failure characteristics(Fig 10).

Fracture angles also have a critical influence on the long-term strength of rock masses. The study found that as fracture angles increase, the long-term strength of rock masses shows a declining trend. Specifically, rock masses with larger fracture angles experience a more pronounced reduction in long-term strength. For example, without freeze-thaw cycling, the long-term strength of rock masses with gentle-gentle fracture angles is 15.778 MPa, while that of rock masses with steep-steep fracture angles is only 4.512 MPa. The increase in fracture angle leads to more stress concentration zones within the rock mass, thereby weakening its long-term strength and reducing its load-bearing capacity.

### 4.3 Interaction effects of freeze-thaw cycles and fracture angles on rock mass failure

The interaction between freeze-thaw cycles and fracture angles presents a particularly complex influence on rock mass failure. Freeze-thaw cycles not only alter the microstructure of rock masses but may also interact with changes in fracture angles to significantly affect creep behavior and long-term strength.

The combined effect of freeze-thaw cycles and fracture angles renders more intricate the creep characteristics of rock masses. Under the influence of freeze-thaw cycles, rock masses with larger fracture angles (e.g., steep-steep fracture angles) exhibit elevated creep rates under loading. Additionally, the creep rate accelerates as the number of freeze-thaw cycles increases. This is because freeze-thaw cycles further advance fracture propagation, enhancing their deformability and thereby intensifying the creep process.

The repeated action of freeze-thaw cycles also exacerbates the failure mechanisms of fractured rock masses. Freeze-thaw cycles not only increase fracture opening and propagation but also promote the development of internal microcracks, aggravating the deterioration process of rock masses with larger fracture angles. For rock masses with large fracture angles, freeze-thaw cycles cause their failure mode to shift from a single tensile or shear failure to a mixed tensile-shear failure. Moreover, the rate and scope of failure significantly increase under the combined influence of freeze-thaw cycles and large fracture angles.

### 4.4 Limitations of the study and future research directions

Although this study systematically explored the effects of freeze-thaw cycles and fracture angles on rock mass failure behavior, several limitations remain. First, the experiments did not simulate more complex environmental conditions, such as variations in water saturation and temperature fluctuations, which could impose further impacts on the mechanical behavior of rock masses. Second, this study was predominantly centered on the creep behavior of fractured rock masses. Future research should delve deeper into the response of fractured rock masses under dynamic loading and examine the effects of freeze-thaw cycles and fracture angles on dynamic failure behavior.

Moreover, with the increasing complexity of geotechnical engineering applications, future studies could incorporate advanced techniques such as acoustic emission monitoring and X-ray computed tomography (CT) to uncover the underlying failure mechanisms and evolutionary trajectories of fractured rock masses under various environmental conditions. This would provide a more robust theoretical basis for the stability analysis of rock masses in engineering practices.

## 5 Conclusion

The impact of fracture angle and freeze-thaw cycles on the creep characteristics and long-term strength of fractured rocks was investigated through freeze-thaw cycling, uniaxial compression, and creep tests under varying conditions. The following conclusions were drawn:

1) Freeze-thaw cycles significantly influence the creep characteristics of rocks. Axial strain in rocks markedly increases with the number of freeze-thaw cycles, and all rock samples exhibit stages of decelerated and constant-rate creep. Under the same stress levels, both instantaneous and creep strains increase gradually with an increase in the number of freeze-thaw cycles, with the growth rate diminishing and stabilizing over time.
2) Different double-fractured rock samples exhibit variations in creep phenomena based on fracture angles. As the fracture angle increases, both instantaneous and creep strains in rocks show noticeable increments. Higher stress levels result in larger deformations in rock samples. At the same stress level, the steady-state creep rate of rocks increases with an increase in fracture angle.
3) Changes in fracture angle affect the failure mode of rock samples. In the creep failure of pre-existing fractured rock samples, all samples exhibit rock bridge breakthrough phenomena. Specifically, gentle-gentle rock samples show a shear failure mode, gentle-steep rock samples exhibit a tensile failure mode, steep-steep rock samples display a tensile-shear mixed failure mode, and intact rock samples undergo a transition from tensile to tensile-shear mixed failure mode with an increase in freeze-thaw cycles. Freeze-thaw cycling intensifies surface cracking and increases the number of failure cracks.
4) Variations in fracture angle and freeze-thaw cycles have a substantial impact on the long-term strength of rocks. The long-term strength of rocks increases with an increase in fracture angle but rapidly decreases with an increase in the number of freeze-thaw cycles.

## Supporting information

S1 data Raw creep-test data for double-fractured rock samples under freeze-thaw cycles. (ZIP)
